# Supplementary material for: Comparing machine and deep learning models for pediatric anxiety classification using structured EHRs and area-based measures of health data
Source: PLoS One. 2026 May 12;21(5):e0324673. doi: 10.1371/journal.pone.0324673 (PMC13166959; doi:10.1371/journal.pone.0324673)
Supplement: S1 Table — (PDF) [file pone.0324673.s001.pdf]

S1 Table . List of ICD codes to determine anxiety patients.

ICD Code	Concept name	Vocabulary id
300	Anxiety state, unspecified	ICD9CM
300.01	Panic disorder without agoraphobia	ICD9CM
300.02	Generalized anxiety disorder	ICD9CM
300.21	Agoraphobia with panic disorder	ICD9CM
300.22	Agoraphobia without mention of panic attacks	ICD9CM
300.23	Social phobia	ICD9CM
309.21	Separation anxiety disorder	ICD9CM
F40.01	Agoraphobia with panic disorder	ICD10CM
F40.02	Agoraphobia without panic disorder	ICD10CM
F40.10	Social phobia, unspecified	ICD10CM
F40.11	Social phobia, generalized	ICD10CM
F41.0	Panic disorder [episodic paroxysmal anxiety]	ICD10CM
F41.1	Generalized anxiety disorder	ICD10CM
F41.9	Anxiety disorder, unspecified	ICD10CM
F93.0	Separation anxiety disorder of childhood	ICD10CM
