# Supplementary material for: Comparing machine and deep learning models for pediatric anxiety classification using structured EHRs and area-based measures of health data
Source: PLoS One. 2026 May 12;21(5):e0324673. doi: 10.1371/journal.pone.0324673 (PMC13166959; doi:10.1371/journal.pone.0324673)
Supplement: S2 Table — (PDF) [file pone.0324673.s002.pdf]

S2 Table . Area-based measures of health (ABMH) data description.

Feature Name	Description	Source
CoI Education	Weighted average of education domain component indicator z-scores, nationally normed	-
CoI Health ENV	Weighted average of health and environment domain component indicator z-scores, nationally normed	-
CoI Social ECON	Weighted average of social and economic domain component indicator z-scores, nationally normed	-
ICE	High income white non-Hispanic households versus low income people of color (not white non-Hispanic) households;	-
Fraction Assisted Income	Fraction of households receiving public assistance income or food stamps or SNAP in the past 12 months	https://geomarker.io/dep_index
Low Food Access Flag	Yes if tract has at least 500 people or at least 33% of the tract population living more than 1 mile from nearest food store in urban areas, or more than 10 miles in rural areas	-
Truck Meters Moving	Average daily number of trucks multiplied by the length of interstates, expressways, and freeways (truck-meters)	https://degauss.org/aadt/
Truck Meters Stop Go	Average daily number of trucks multiplied by the length of arterial roads (truck-meters)	https://degauss.org/aadt/
Vehicle Meters Moving	Average daily number of vehicles multiplied by the length of interstates, expressways, and freeways (vehicle-meters)	https://degauss.org/aadt/
Vehicle Meters Stop Go	Average daily number of vehicles multiplied by the length of arterial roads (vehicle-meters)	https://degauss.org/aadt/
Fraction Poverty	Fraction of households with income below poverty level within the past 12 months	https://github.com/geomarker-io/hh_acs_measures/releases
Fraction Insured	Fraction of population with health insurance (available from 2012 onwards only)	https://github.com/geomarker-io/hh_acs_measures/releases
Fraction SNAP	Fraction of households receiving food stamps/SNAP in the past 12 months	https://github.com/geomarker-io/hh_acs_measures/releases
Fraction FAM No Spouse	Single householder is male or female household, with no spouse present	https://github.com/geomarker-io/hh_acs_measures/releases
Fraction Built BF 1970	Fraction of Housing Units Built Before 1970	https://github.com/geomarker-io/hh_acs_measures/releases
Fraction Vacant	Fraction of Housing Units that are Vacant	https://github.com/geomarker-io/hh_acs_measures/releases
EVI 2500	Average enhanced vegetation index within a 2500 meter buffer radius	https://degauss.org/greenspace

Abbreviations: CoI = Child Opportunity Index; ENV = Health & Environment domain; ECON = Social & Economic domain; ICE = Index of Concentration at the Extremes; SNAP = Supplemental Nutrition Assistance Program; AADT = Annual Average Daily Traffic; EVI = Enhanced Vegetation Index; BF = Built Before; FAM = Family.
