# Supplementary material for: Comparing machine and deep learning models for pediatric anxiety classification using structured EHRs and area-based measures of health data
Source: PLoS One. 2026 May 12;21(5):e0324673. doi: 10.1371/journal.pone.0324673 (PMC13166959; doi:10.1371/journal.pone.0324673)
Supplement: S1 Appendix — Provide extensive information about the time-dependent and static features in the dataset. (PDF) [file pone.0324673.s003.pdf]

S1 Appendix

Structured EHR Data

Diagnosis Codes

Diagnosis codes are encoded in the EHR using the International Classification of Diseases (ICD). The hospital transitioned from ICD-9 to ICD-10 in late 2015. To maintain consistency of coding over time, we used the General Equivalence Mappings (GEM) to convert ICD-9 to ICD-10 codes, using the R package "touch" (`https://cran.r-project.org/web/packages/touch/index.html`). Subsequently, all resulting ICD-10 diagnosis codes were then mapped to Clinical Classification Software Refined (CCSR) categories, maintained by the Agency for Healthcare Research and Quality (AHRQ) and the Healthcare Cost and Utilization Project (H-CUP) (`https://hcup-us.ahrq.gov/toolssoftware/ccsr/DXCCSR-Reference-File-v2023-1.xlsx`). Within this coding framework, numerical values denote the frequency of ICD codes mapped to the respective CCSR categories in the Electronic Health Record (EHR) for the specified time frame. A value of 0 signifies that at least one diagnosis was recorded within the bin, yet it did not align with any CCSR category. Conversely, NaN indicates the absence of any recorded diagnosis data for the given time period.

Procedure Codes

The EHR uses over 24,000 unique Current Procedural Terminology (CPT) codes. To reduce the dimensionality and systematize the data, these were mapped to the Clinical Classifications Software (CCS) for Services and Procedures categories maintained by the Agency for Healthcare Research and Quality (AHRQ) (`https://www.hcup-us.ahrq.gov/toolssoftware/ccs_svcsproc/ccscpt_downloading.jsp`). Additionally, we categorized each CPT, Healthcare Common Procedure Coding System (HCPCS), and ICD-10 Procedure Coding System (ICD-10-PCS) code by the recommended classification at the time of the procedure. This meticulous categorization yielded an impressive coverage of approximately 99% of all procedure codes, resulting in 246 final categories of procedures. Our coding framework encodes the frequency of occurrence for each category within a specified time-bin. A value of 0 signifies the absence of occurrences for the category within the time-bin. Conversely, null values represent instances where no visits were recorded during the specified time period.

Medication Codes

We used Epic Clarity's pharmaceutical class and sub-class categorizations (licensed from Medi-Span (`https://www.wolterskluwer.com/en/solutions/medi-span`) to categorize patients' medication exposures. We identified 94 pharmaceutical classes and 764 pharmaceutical sub-classes in our dataset. The frequency of occurrences for each medication sub-class within a specified time-bin was encoded within our coding framework. A value of 0 indicates the absence of occurrences for the sub-class within the time-bin. Conversely, null values denote instances where no medication occurrences were recorded during the specified time period.

Visit Metadata

The following features identify information about the types of visits/encounters and healthcare providers that a patient sees in a time bin. These are all frequency-encoded for the number of visits of each type that occurred within a time bin, with 0 indication that no visit of that type occurred, and a NaN/null indicating that no visit at all occurred. For each, any categorizations representing less than 0.1% of all visits are mapped to Other.

- **Encounter Type:** "Encounter type" categorizes the type of visit. This includes classifications like office visits, emergency room visits, telehealth, phone encounter, etc. There are a total of 130 used encounter type categories.
- **Provider Type:** "Provider type" categorizes the type of healthcare provider seen during an encounter. This includes classifications like physicians, nurses, therapists, etc. There are a total of 65 provider type categories.
- **Care Site:** "Care site" contains the department and physical location of the visit. This includes things like Orthopaedics or Cardiology, and the specific campus or building it was located in. There are 467 distinct care sites used, including Other.

- **Place of Service:** "Place of service" indicates a medical specialty within the Care Site/department. There are 77 distinct place of service values used, including Other.

Hospitalization

We conducted an analysis to determine the length of hospitalization for inpatient hospital encounters. This calculation was performed by extracting admission and discharge dates and subsequently computing the duration of each hospitalization episode. The resultant dataset provides the number of hospitalization days aggregated into time-bins, effectively covering the entire hospitalization time frame.

Measurements

Our features from structured EHR recorded various measurements, including Body Mass Index (BMI), height, weight, blood pressure, and heart rate. To ensure robust analysis, all measurements' percentiles were meticulously computed in alignment with the standards the Centers for Disease Control and Prevention (CDC) set forth. Specifically, measurements were considered valid for individuals aged 24 months or older. Comprehensive calculations were performed for BMI percentiles, taking into account age, sex, height, and weight. Similarly, height and weight measurements were analyzed by computing z-scores relative to individuals' age, height, and sex. In the case of blood pressure, we employed a nuanced approach by calculating age- and gender-specific percentiles for both systolic and diastolic blood pressure, factoring in age, gender, and height parameters. In our dataset, NaN values were utilized to signify instances where no measurement data was recorded for the respective date, ensuring clarity and integrity in our analysis.

Static Features

- **Allergies:** We employed Natural Language Processing (NLP) techniques to categorize allergens listed in the EHRs into three distinct categories: Food, Medications, and Environment. These categories were encoded with frequencies per individual, representing the occurrence count per person within specified time intervals. A value of 0 denotes the absence of occurrences for a category in the given time-bin.
- **Details:** Birth history variables, including gestational age in weeks, birth weight, and birth height, were incorporated at the individual level. Additionally, three supplementary fields were included:
  - 'is interpreter needed processed': Categorized as 1 for 'Yes' and 0 for 'No'.
  - 'Preferred language processed': Language preferences were standardized, allowing only 'English', 'Spanish', or marking other languages as 'Unknown'.
  - 'Gender identity matches to gender': Aligned with set rules; if gender identity exactly matches gender, the value is set to 1; otherwise, it is set to 0. Null values are assigned when the source value is null or 'choose not to disclose'.
- **Family History:** Patient-level family medical history was derived from patient notes, extracting relationship and disease types. Diseases falling under mental health conditions were categorized into six distinct categories: psychiatric disorders, substance abuse, sexual/verbal abuse, autism, attention-deficit/hyperactivity disorder, and developmental disorder. Patients meeting these criteria were filtered based on relationships identified as mother, father, sister, half-sister, brother, or half-brother. This data was frequency encoded, with NaNs indicating no family medical problems matching the specified mental health-based filters. Additionally, 'Neg Hx' may be utilized in certain instances to signify the absence of a specific disorder within the family history.
- **Demographics:** Demographic information encompasses race, ethnicity, and sex. Races and ethnicities were encoded with unique positive integer values, while sex was encoded with 1 for males and 0 for females.
