# Supplementary material for: Comparing machine and deep learning models for pediatric anxiety classification using structured EHRs and area-based measures of health data
Source: PLoS One. 2026 May 12;21(5):e0324673. doi: 10.1371/journal.pone.0324673 (PMC13166959; doi:10.1371/journal.pone.0324673)
Supplement: S2 Appendix — Discuss the specifics of the ML- and DL-based classification models that are applied. (PDF) [file pone.0324673.s004.pdf]

S2 Appendix

Classification Models

Machine Learning-based Models

- **Logistic Regression:** Logistic regression (LR) is a conventional statistical model commonly employed for binary classification applications. The model posits a Bernoulli distribution between the input feature matrix, X, and the output target class, y. The input feature matrix X is a vector derived from features extracted from structured EHR or ABMH data.
- **Decision Tree:** Decision Trees (DT) is a supervised learning model utilized for both classification and regression tasks. The objective is to develop a model that can forecast the value of a specific variable by acquiring basic decision rules from the characteristics of the data.
- **Random Forest:** Random forest (RF) [30] is a popular machine-learning technique that aggregates the predictions of numerous decision trees to get a final output. Its user-friendly interface and adaptability have driven its widespread use since it can effectively address both classification and regression tasks.
- **K-Nearest Neighbor:** The K-Nearest Neighbors (KNN) algorithm is a widely used ML method for classification and regression applications. It is based on the concept that similar data points will likely have corresponding labels or values, allowing each data point to be grouped.
- **Extreme Gradient Boosting:** Extreme Gradient Boosting (XGBoost) [31] is a gradient-boosted decision tree model commonly utilized in machine learning and data mining competitions for classification and regression tasks. XGBoost is a gradient-boosting algorithm known for its excellent efficiency and flexibility, specifically developed to prioritize speed and performance. XGBoost constructs trees in a unique and efficient manner. It does this by incrementally using the gradient statistics of the binary classification loss, which are derived from the input feature matrix, X.

Deep Learning-based Models

- **Long Short-Term Memory:** Long Short-Term Memory (LSTM) [33] is a specific type of RNN model that can recognize and learn the importance of order in sequence prediction tasks. LSTM is more advanced than standard RNNs because it includes an input, forget, and output gate. These gates can selectively preserve or eliminate information from the hidden state to enhance the retention of long-term dependencies in the data.
- **Gated Recurrent Unit:** The Gated recurrent unit (GRU) [32] is designed to address the issue of the vanishing gradient problem inherent in a traditional RNN model. GRU can be viewed as a variant of LSTM because of their similar design and comparable performance in some scenarios. GRU addresses the vanishing gradient issue in a normal RNN by employing update and reset gates. These two vectors determine the information that will be transmitted to the output. They can retain historical information without temporal distortion or filtering out extraneous data for forecasting.
- **Reverse Time Attention:** Reverse Time Attention (RETAIN) [18] is an RNN-based model that resolves the limitation of general RNN-based models such as GRU and LSTM. RNN-based models are effective in sequential inputs. However, it is difficult to tell what causes the outcome as all inputs are accumulated at the last layer. RETAIN adopts attention technique for sequential data to build a predictive model for healthcare.
- **Diagnosis Prediction Model:** The Diagnosis Prediction Model (Dipole) [19] is an RNN-based model that uses the attention mechanism to make sequential predictions of clinical events. Dipole employs an attention-based bidirectional RNN to examine the relationships between past and future visits and produce predictions for diagnosis.
