# Supplementary material for: Comparing machine and deep learning models for pediatric anxiety classification using structured EHRs and area-based measures of health data
Source: PLoS One. 2026 May 12;21(5):e0324673. doi: 10.1371/journal.pone.0324673 (PMC13166959; doi:10.1371/journal.pone.0324673)
Supplement: S3 Appendix — Discuss the hyperparameter tuning procedure, as well as the hyperparameters selected for ML- and DL-based models. (PDF) [file pone.0324673.s005.pdf]

S3 Appendix

Hyperparameter Tuning

For the ML-based models, we use GridSearchCV from scikit-learn [46] for hyperparameter tuning. Here is the list of hyperparameters used for tuning the ML-based models. For the LR, we use Penalty = $[l1, l2]$, C = $[1.0, 0.5, 0.1]$, and solver = $[liblinear]$. For DT, we use criterion = $[gini, entropy]$, min_sample_leaf (msl) = $[1, 2, 3, 4, 5, 6]$, max_depth (md) = $[1, 2, 3, 4, 5, 6]$, and min_sample_split (mss) = $[2, 3, 4, 5, 6]$. For RF, we use min_samples_leaf (msl) = $[1, 2, 3, 4, 5, 6]$, max_depth (md) = $[1, 2, 3, 4, 5, 6]$, and min_samples_split (mss) = $[2, 3, 4, 5, 6]$. For KNN, we use n_neighbors (nn) = $[1, 2, 3, 4, 5, 6]$, weights = $[uniform, distance]$, and metric = $[euclidean, manhattan]$. For XGBoost, we use learning_rate (lr) = $[0.1, 0.2, 0.3]$, max_depth (md) = $[1, 2, 3, 4, 5, 6]$, min_child_weight (mcw) = $[1, 2]$, subsample = $[1.0, 0.5, 0.1]$, and n_estimators (ne) = $[50, 100, 150]$. Selected hyperparameters for ML-based models are summarized in S3 Appendix Table 1.

S3 Appendix Table 1. Selected hyperparameters for ML-based models.

	**LR**		**DT**				**RF**			**KNN**		**XGBoost**				
**Age group**	**C**	**penalty**	**criterion**	**md**	**msl**	**mss**	**md**	**msl**	**mss**	**metric**	**nn**	**lr**	**md**	**mcw**	**ne**	**subsample**
2	0.5	L1	gini	5	6	2	4	3	2	manhattan	5	0.1	6	2	50	1
3	0.1	L1	gini	3	3	2	5	1	2	euclidean	6	0.1	2	1	50	0.5
4	0.5	L1	entropy	6	6	2	6	1	2	manhattan	6	0.1	4	2	150	0.1
5	0.1	L1	entropy	6	5	2	6	4	2	manhattan	6	0.1	4	2	100	0.5
6	1	L1	entropy	6	6	2	6	1	2	euclidean	6	0.2	1	1	50	1
7	0.5	L1	entropy	6	6	2	6	5	2	manhattan	6	0.2	1	2	100	0.5
8	0.1	L1	entropy	5	2	2	6	3	2	euclidean	6	0.3	2	1	50	1
9	0.1	L1	entropy	5	3	2	6	1	5	manhattan	6	0.1	5	2	50	0.5
10	0.1	L1	gini	6	6	2	6	4	2	euclidean	6	0.1	2	2	150	0.1
11	0.5	L1	gini	3	6	2	6	1	2	manhattan	6	0.1	1	1	150	0.1
12	0.1	L1	entropy	6	6	2	6	1	2	manhattan	6	0.1	3	1	100	1
13	0.1	L1	entropy	3	1	2	6	1	4	manhattan	6	0.2	1	1	100	1
14	0.1	L1	gini	4	2	2	6	1	6	euclidean	6	0.1	1	1	150	0.5
15	0.1	L1	gini	2	1	2	6	5	2	euclidean	6	0.2	2	2	150	0.1
16	0.1	L1	gini	4	3	2	6	3	2	euclidean	6	0.1	1	1	150	1
17	0.1	L1	gini	4	1	2	5	1	5	manhattan	6	0.2	2	2	100	0.5
18	0.1	L1	gini	4	1	2	5	1	3	euclidean	6	0.1	2	1	100	1
19	1	L1	gini	6	6	2	6	1	3	manhattan	6	0.1	1	1	100	0.5
20	0.1	L2	gini	4	5	2	6	1	6	euclidean	6	0.1	4	1	50	1
21	0.1	L1	gini	5	6	2	6	3	2	manhattan	6	0.1	4	1	50	1

Notes: For the solver in Logistic Regression (LR), `liblinear` was selected, and uniform weights were used for KNN across all age groups.
Abbreviations: md = minimum depth; msl = minimum samples per leaf; mss = minimum samples per split; nn = number of neighbors; lr = learning rate; mcw = minimum child weight; ne = number of estimators.

For the DL-based models, we use grid search for hyperparameter tuning. Here is the list of hyperparameters used for tuning the DL-based models. For LSTM, batch_size (bs) = $[16, 32, 64]$, learning_rate (lr) = $[0.001, 0.0001]$, hidden_dimension (hd) = $[16, 32, 64]$, and number_of_layers (n_layer) = $[1, 2, 3]$. For GRU, we use batch_size (bs) = $[16, 32, 64]$, learning_rate (lr) = $[0.001, 0.0001]$, hidden_dimension (hd) = $[16, 32, 64]$, and number_of_layers (n_layer) = $[1, 2, 3]$. For RETAIN, we use batch_size (bs) = $[16, 32, 64]$, learning_rate (lr) = $[0.001, 0.0001]$, and dropout = $[0.1, 0.3, 0.5]$. For Dipole, we use batch_size (bs) = $[16, 32, 64]$, learning_rate (lr) = $[0.001, 0.0001]$, hidden_dimension (hd) = $[16, 32, 64]$, embedding_dimension (ed) = $[16, 32]$, and dropout = $[0.1, 0.5]$. Selected hyperparameters for DL-based models are summarized in S3 Appendix Table 2.

S3 Appendix Table 2. Selected hyperparameters for DL-based models.

	LSTM				GRU				RETAIN			Dipole				
**Age group**	**bs**	**lr**	**hd**	**n_layer**	**bs**	**lr**	**hd**	**n_layer**	**bs**	**lr**	**dropout**	**bs**	**lr**	**hd**	**ed**	**dropout**
2	16	0.0001	64	1	64	0.0001	64	1	64	0.001	0.3	64	0.0001	32	16	0.1
3	16	0.0001	64	1	32	0.0001	32	1	64	0.0001	0.1	16	0.0001	32	16	0.1
4	64	0.0001	64	1	64	0.0001	64	1	32	0.001	0.3	16	0.0001	64	16	0.1
5	64	0.0001	64	1	32	0.0001	64	3	32	0.001	0.5	64	0.0001	16	16	0.1
6	64	0.0001	32	1	32	0.0001	64	1	32	0.001	0.5	64	0.0001	64	16	0.1
7	32	0.0001	64	1	16	0.0001	64	1	16	0.001	0.1	64	0.0001	16	16	0.1
8	64	0.0001	32	1	64	0.0001	16	1	16	0.0001	0.5	16	0.0001	32	16	0.1
9	32	0.0001	32	1	64	0.0001	32	3	64	0.001	0.5	32	0.0001	16	16	0.1
10	32	0.0001	64	1	16	0.0001	16	2	64	0.001	0.5	16	0.0001	16	16	0.1
11	16	0.0001	64	1	32	0.0001	32	3	16	0.001	0.3	64	0.0001	16	16	0.1
12	32	0.0001	32	1	16	0.0001	16	1	64	0.001	0.5	32	0.0001	64	16	0.1
13	64	0.0001	32	1	16	0.0001	32	3	32	0.001	0.5	64	0.0001	16	16	0.1
14	64	0.0001	32	1	16	0.0001	64	1	16	0.001	0.5	64	0.0001	32	16	0.1
15	16	0.0001	32	1	16	0.0001	64	1	64	0.001	0.3	16	0.0001	64	16	0.1
16	32	0.0001	64	1	16	0.0001	64	3	16	0.0001	0.3	64	0.0001	16	16	0.1
17	64	0.0001	64	1	64	0.0001	32	1	32	0.001	0.5	64	0.0001	32	16	0.1
18	16	0.0001	16	1	64	0.0001	64	1	64	0.0001	0.3	64	0.0001	16	16	0.1
19	16	0.0001	64	1	64	0.0001	64	1	64	0.001	0.3	16	0.0001	64	16	0.1
20	32	0.0001	64	1	32	0.0001	32	1	32	0.001	0.3	32	0.0001	32	16	0.1
21	16	0.0001	32	1	16	0.0001	64	1	64	0.001	0.1	64	0.0001	32	16	0.1

Abbreviations: bs = batch size; lr = learning rate; hd = hidden dimension size; n_layer = number of layers; ed = embedding dimension size.
