# Supplementary material for: Comparing machine and deep learning models for pediatric anxiety classification using structured EHRs and area-based measures of health data
Source: PLoS One. 2026 May 12;21(5):e0324673. doi: 10.1371/journal.pone.0324673 (PMC13166959; doi:10.1371/journal.pone.0324673)
Supplement: S4 Appendix — Present the additional results using various evaluation metrics (accuracy, NPV, F1 score, and AUPRC) for both EHR and EHR+ABMH features of ML- and DL-based models. (PDF) [file pone.0324673.s006.pdf]

S4 Appendix

Additional Results

This section shows the performance of evaluation metrics that is not discussed which are accuracy, NPV, F1 score, and AUPRC. S4 Appendix Fig 1 and 2 shows all the results on different evaluation metrics discussed for both *EHR* and *EHR+ABMH* features of ML- and DL-based models, respectively. The figure display the mean of 1000 bootstrapping iterations at each data point, while the colored region represents the 95% confidence interval (CI). However, CIs are tight; therefore, they are not visible in some places on the figure. For the ML-based models, S4 Appendix Fig 1(a) and S4 Appendix Fig 1(b) show the accuracy of ML-based models of all the age groups utilizing *EHR* and *EHR+ABMH* features, respectively. S4 Appendix Fig 1(c) and S4 Appendix Fig 1(d) show the NPV, S4 Appendix Fig 1(e) and S4 Appendix Fig 1(f) show the F1 score, and S4 Appendix Fig 1(g) and S4 Appendix Fig 1(h) show the AUPRC. For the DL-based models, S4 Appendix Fig 2(a) and S4 Appendix Fig 2(b) show the accuracy of ML-based models of all the age groups utilizing *EHR* and *EHR+ABMH* features, respectively. S4 Appendix Fig 2(c) and S4 Appendix Fig 2(d) show the NPV, S4 Appendix Fig 2(e) and S4 Appendix Fig 2(f) show the F1 score, and S4 Appendix Fig 2(g) and S4 Appendix Fig 2(h) show the AUPRC.

S4 Appendix Fig 1. Accuracy, NPV, F1 score, and AUPRC results of ML-based models.

S4 Appendix Fig 2. Accuracy, NPV, F1 score, and AUPRC results of DL-based models.
